# Prevalence of occupational injuries and associated factors among small scale industry Workers in Harar Town, eastern Ethiopia

**DOI:** 10.3389/fpubh.2025.1532799

**Published:** 2025-02-24

**Authors:** Desi Debelu, Sina Temesgen Tolera, Negga Baraki, Dechasa Adare Mengistu

**Affiliations:** College of Health and Medical Sciences, Haramaya University, Dire Dawa, Ethiopia

**Keywords:** injury, occupational injury, small-scale industry, Harar town, work place, Ethiopia

## Abstract

**Background:**

Currently, small-scale industries pose significant risks for occupational injuries, particularly in developing countries, including Ethiopia. Despite this problem, there is limited evidence on the prevalence of occupation-related injuries in small-scale industries that can be utilized for proposing interventions. Therefore, this study aims to determine the prevalence of occupational injuries and associated factors among small-scale industry workers in Harar town, Ethiopia.

**Methods:**

A cross-sectional study employing a quantitative analysis was conducted in Harare town, Eastern Ethiopia, from March 12, 2023, to June 1, 2023. The source of population was all small-scale industry workers in Harar town, while the study population was workers working in selected small-scale industries. The sample size was determined based on the previous study finding reported 35.98% prevalence of occupational injuries. A simple random sampling method was employed to select the study participant, resulting in the inclusion of 639 participants in the study. Data were collected using pretested questionnaires and observational checklists, covering sociodemographic, occupational injuries, occupational health and safety practices, work environment, and behavioral related factors. The data were analyzed via SPSS version 22, and a *p* value of <0.05 was considered the cut-off point for statistical significance in multivariate analysis.

**Results:**

Out of the 639 small-scale industry workers included in the current study, 634 provided a response, resulting in a 99.2% response rate. Among these workers, 417 workers (65.8%) reported exposure to occupational injuries at least once in their careers, of which 223 (35.2%) were exposed to injuries in the last 12 months. The most commonly injured body parts were the hands (34.11%), legs (29.13%), and fingers (26.69%). The study found a significant associations between occupational injuries and type of industry (AOR: 2.41, 95% CI: 1.07, 5.46), educational status (AOR: 2.49, 95% CI: 1.01, 2.83), training (AOR: 1.4, 95% CI: 1.35, 3.22), working space (AOR: 4.6, 95% CI: 2.62, 7.51), and workload (AOR: 2.88, 95% CI: 2.78, 11.64).

**Conclusion:**

More than six out of 10 workers experienced injuries during their careers, with more than one-third being injured in the last 12 months. Workers with lower educational status, have no training, working in poor working conditions, and with higher workloads are at high risk of exposure. This highlights the need for improving occupational health and safety practices, including tailored training and supportive supervision to reduce injury in small-scale industries.

**Originality/value:**

The findings from this study will be important for concerned bodies aiming to improve the occupational health and safety practices in small-scale industries and enhance the safe practices by addressing the safety culture in the working environment and policy making process.

## 1 Introduction

Rapid industrial, scientific, and social development, coupled with the use of non-renewable natural resources, has significantly increased occupational risks worldwide (1, 2). Small-scale industries (SSIs), including woodworking, metalworking, concrete block, and manufacturing industries, are among the highest-ranked SSIs in terms of risks for occupational injuries (3, 4).

According to global estimates from the International Labor Organization (ILO), poor occupational health and safety contribute to more than 2.3 million deaths annually. Among these deaths, approximately 350,000 result from fatal accidents, whereas approximately 2 million are due to fatal work-related diseases. Additionally, more than 313 million workers experience nonfatal occupational accidents that lead to serious injuries and significant absences from work (5).

In developed countries, occupational health services are available for about 20–50% of the workers, whereas in developing countries, they cover only 5–10% of workers. Despite this disparity, the prevalence of injuries is lower in high-income countries than in middle- and low-income countries (LMICs). For example, fatality and accident rates in Sub-Saharan Africa are significantly higher, accounting for 21 and 16,012 per 100,000 workers, respectively (6, 7).

Furthermore, workdays lost due to occupational injuries represent a significant economic challenge globally. The World Health Organization (WHO) estimates that the economic loss from work-related injuries and diseases accounted for 4% of the global GDP. In 2010 alone, over 313 million workers experienced nonfatal injuries that led to at least 4 days of absence from work (8, 9). Additionally, workers who suffer from occupational injuries may also face social and psychological trauma (10). Therefore, application of a positive psychological measure can significantly improve the implementation of safety measures through incorporating psychological related factors into policy-making or frameworks in the industry (11).

In Ethiopia, occupational injuries are major causes of mortality and morbidity among workers working in small-scale enterprises (12, 13). Many SSIs in Ethiopia lack sufficient safety materials, guidelines, supervision, and policies to prevent workplace injuries and promote worker health. Furthermore, there is a lack of adequate data on the prevalence of work-related injuries due to limited national and local information (14). Some of the studies conducted in Ethiopia were targeted at prevalence of occupational injuries specific to body parts such as ocular injury (15), while the others targeted at workers working in specific industries such as metal manufacturing industry (16), small-scale woodwork industry workers (14, 17), and building construction workers (13). However, this study addresses the prevalence of occupational injuries in different types of small scale industries and body parties, which provide comprehensive evidence that can be utilized by the concerned bodies to take action.

Furthermore, in Harare town, there is no data or evidence on the prevalence of occupational injuries among. Therefore, this study aims to determine the prevalence of occupational injuries and associated factors among small-scale industries workers in the Harare town. The findings from this study are used to understand the extent of the problem and the conditions that lead to injuries, which can help in developing targeted interventions and improving workplace safety for these workers.

## 2 Literature review (overview)

Currently, the workplace influences the health and safety, as well as the productivity of workers at different levels (18). Industry workers are exposed to huge stressors in their working environment. This exposure is prolonged due to unusual work schedules in many industries (19). Among these industries, small-scale industries, such as construction industries, are facing high safety risks, requiring effective safety leadership to reduce accidents and improve performance (20). According to the ILO report in 2023, more than 2.93 million deaths and 395 million non-fatal work-related injuries and illnesses occurred from occupational accidents and diseases each year around the world. Furthermore, the economic cost of these occupational-related deaths, injuries, and illnesses is estimated at 361 billion dollars each year (21).

Therefore, the hazardous nature of different industries’ activities across the world and higher in the developing countries has led to concerns about the occupational health and safety of the workers. It highlighted the critical need for adopting safe work methods or environments (22, 23). Health and safety is of paramount importance, especially for different organizations and individuals operating in high-risk industrial areas. Therefore, there should be adequate workplace safety management and accident prevention through proper design of working conditions and implementation of basic protection (24, 25).

According to a cross sectional study conducted in Bale Zone, southeast Ethiopia, the prevalence of occupational injury among SSI workers throughout the lifetime, and in the last 1 year was 43.2, and 30%, respectively (26). Another cross sectional study conducted in Ambo town, Ethiopia in 2021, among small scale enterprise workers in 2022 revealed that the prevalence of occupational injuries in the last 1 year was 39.5% (95% CI: 35–44) (27). According to a cross sectional study conducted in Mekele City, Ethiopia in 2013, among SSI workers, the use of personal protective equipment (PPE) [AOR = 3.43, 95%CI: 2.39–4.94] was significantly associated with occupational injury (28). Moreover, a cross sectional study conducted in ambo town in 2021 reported that there was an association between occupational injury among small scale industry workers and not using PPE (AOR = 1.55, 95% CI: 1.03–2.87) (27).

We remain in need of further discussion and application of comprehensive models that integrate the role of cognitive challenges, emotional states, and organization of tasks, work stress, health, and work environmental factors (25). Therefore, timely identification of the gap and solutions for the potential risk factors is critical for the health and safety, as well as for the productivity of the workers. Because improving workers health and safety is paramount for industries, including in reducing the shortage of skilled manpower, strains on supply, and medical costs related to this problem (18).

Therefore, industries need to tailor their workplace safety measures to address the diverse needs of their workforce. In light of this study’s findings, this study aimed to determine the prevalence of occupational injuries and associated factors among small-scale industries workers expected to have a high risk of occupational injuries (11).

### 2.1 Conceptual framework

**Figure 1:**
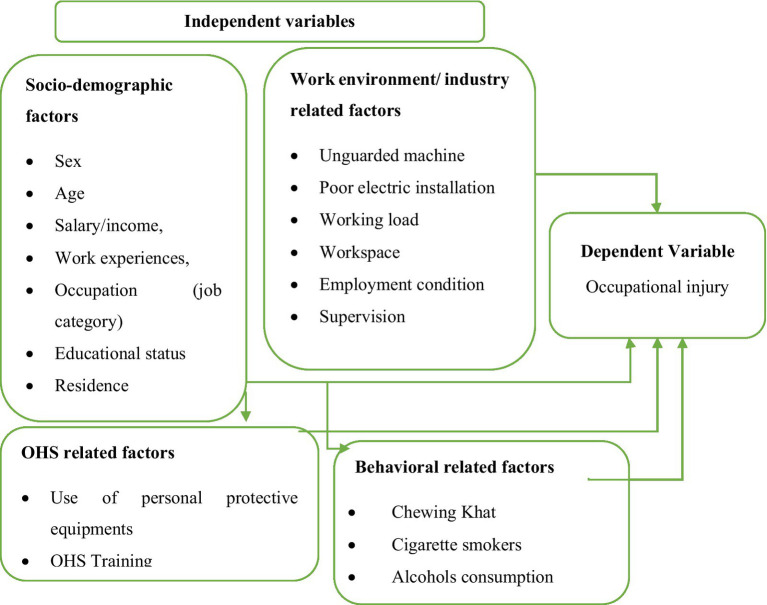
Conceptual framework used to assess factors associated with occupational related injury among small-scale industry workers (Developed by the investigator).

A conceptual framework showing the interaction between dependent variables (occupational related injury) and independent variables (Figure 1).

## 3 Materials and methods

### 3.1 Study area and period

The study was conducted in Harar town, Eastern Ethiopia, from March 12, 2023 to June 1, 2023. Harar town is located approximately at 520 km from Ethiopia’s capital city, Addis Ababa, and lies at a latitude of 9°19′N and a longitude of 42°7′E. According to the 2015/2016 census, Harari Regional state has a population of 255,690. Harar town has 1,025 SSIs and 3,151 workers in industries such as woodworking, metalworking, construction, and concrete.

### 3.2 Study design

A cross-sectional study design with quantitative data analysis methods was employed to assess the prevalence of occupational injuries and associated factors among scale industry workers in Harare town.

### 3.3 Source and study population

All small scale industry workers working in Harare town were a source population, while small scale workers working in the selected industries; woodworking, metalworking, construction, and concrete/stone industries were a study population.

### 3.4 Inclusion and exclusion criteria

All small-scale industry workers in Harare town who were available during the study period were included in the research. Workers who were on annual leave or who were seriously ill during the data collection period were excluded from the study.

### 3.5 Sample size determination and sampling techniques

The sample size for the study was calculated using a prevalence rate of 35.98% from a previous study (29), with a 5% margin of error, a 95% confidence interval, and a design effect of 2. Considering a 10% nonresponse rate (58 participants), the total sample size required was 639.

**Figure 2:**
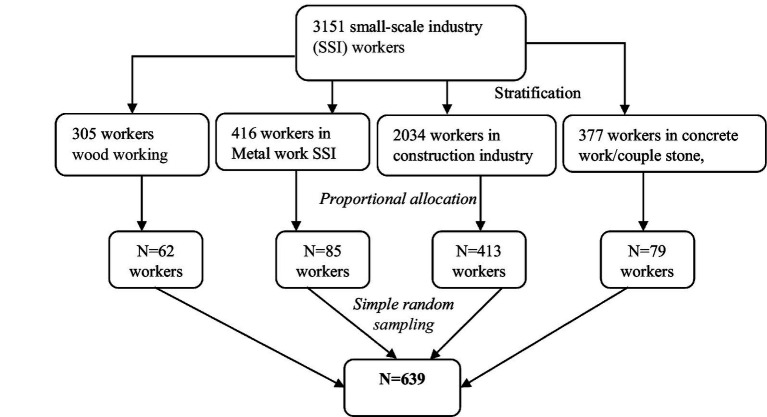
Sampling technique/procedures to be employed in selecting small scale industry workers found in Harare Town, 2023.

Study participants were selected via a simple random sampling technique after proportional allocation to the required sample size for each SSI category. The SSIs were first stratified on the basis of their type, and then participants were randomly selected from each stratum to ensure representative sampling (Figure 2).

### 3.6 Data collection method

Four BSc holders’ health professionals were involved in data collection to collect all the data required for the study under the guidance of one superior and principal investigator during data collection. A pre-tested structured questionnaire and observational checklist were used to collect data on the following section; sociodemographic characteristics (10 questions), occupational injuries (8 questions), OHS-related factors (10 questions), work environment factors (6 questions), and behavioral-related factors (6 questions). The questionnaire was adapted from the WHO, OSHA, and Ethiopian national guidelines. Moreover, an observational checklist was applied to triangulate the quantitative data. The questionnaire was subsequently translated into the local languages of the study participants, Afan Oromo and Amharic.

### 3.7 Study variables

Dependent variable: Occupational-related injuries.

Independent variables:

- Sociodemographic aspects (sex, age, income, work experience, occupation, risk perception, educational status, and SSIs ownership)
- OHS-related factors (personal protective equipment, OHS training, and the availability of PPE)
- Work environment conditions (including lighting, workload, workspace, and supervision)
- Behavioral factors (chewing khat, cigarette smoking, and alcohol consumption).

### 3.8 Data quality control

Before data collection, the questionnaire was pretested in selected SSIs in Haramaya town, Eastern Ethiopia, to assess the clarity, sequence, and relevance of the questions and to estimate the time required for each respondent. Data collectors and supervisors received 1 day of training on the principles, ethics, procedures, and tools used in the study. The principal investigator closely monitored the data collection process, and all completed questionnaires were reviewed daily for consistency and completeness.

### 3.9 Data processing and analysis

The data were entered, cleaned, and analyzed using SPSS version 22 software. Descriptive statistics, including frequencies and proportions, were used to summarize the data. Bivariate and multivariate logistic regression analyses were employed to assess associations between dependent and independent variables. The crude odds ratio (COR) and adjusted odds ratio (AOR) with 95% confidence intervals were used to describe the relationships between risk factors and occupational injury exposure. Finally, the results are presented in text, tables, and figures. A *p* value of 0.2 was considered as the cut-off for statistical significance in the bivariate analysis, whereas a p value of 0.05 was used for multivariate analysis.

## 4 Results

### 4.1 Sociodemographic characteristics

The survey included 639 small-scale industry workers, of whom 634 provided responses, resulting in a high response rate of 98.6%. Among the respondents, 84.2% were male, and the mean age was 29.04 ± 6.02 years. A significant majority of the workers (93.7%) were temporarily employed and worked more than 8 h each day. Among the study participants, 43.4% had attended elementary school, and 4.6% had no formal education. The income for 76% of the workers ranged from 36.5 to 72.1 USD, with a mean of 50.4 ± 26.6 USD. In terms of the number of years of service in the SSIs, 44.6% had 4–6 years of experience, whereas 38.5% had 3 or fewer years (Table 1).

**Table 1:** Sociodemographic characteristics of small-scale industry workers in Harare town, Ethiopia, 2023.

Variables (*n* = 634)	Categories	Frequency	Percent
Types of SSIs	Concrete	60	9.5
	Construction	210	33.1
	Metalwork	189	29.8
	Woodwork	175	27.6
Gender	Male	534	84.2
	Female	100	15.8
Age in years	18–29	375	59.1
	30–39	219	34.5
	40–49	40	6.30
Marital status	Single	391	61.7
	Married	215	33.9
	Widowed and divorced	28	4.40
Educational status	Have no formal education	29	4.60
	Elementary school	275	43.4
	Secondary school	220	34.7
	Diploma and above	110	17.4
Employment condition	Permanent	40	6.3
	Temporally	594	93.7
Working hours	≤8 h	40	6.3
	>8 h	594	93.7
Income (In USD)	<=36.04	73	11.5
	36.5 to 72.1	482	76.0
	72.2–108.11	37	5.8
	>108.11	42	6.6
Service year of SSI	<=3	244	38.5
	4–6	283	44.6
	7–9	68	10.7
	> = 10	39	6.2
Service year of employee	≤3	466	73.5
	4–6	132	20.8
	7–9	36	5.7

ETB, Ethiopian Birr; SD, Stand and Deviation; USD, United States Dollar; one USD = 55.5 Ethiopian Birr.

### 4.2 Occupational health and safety practice

Among the 634 SSIs workers surveyed, 601 (94.8%) did not have personal protective equipment (PPE), such as gloves, gowns, face shields, or earplugs. Gowns and safety clothing were unavailable for 579 (91.3%) of the SSIs. Only 27 (4.26%) of the workers were reported having adequate PPE. Additionally, 49 (7.7%) of the workers had received training on occupational health and safety. Adequate lighting and workspace were present in 516 (81.4%) and 490 (77.3%) of the SSIs, respectively. Materials were not properly secured in 583 (92.0%) of the SSIs, and only 7 (1.1%) of the SSIs had a first-aid kit (Table 2).

**Table 2:** Occupational health and safety practices among small-scale industry owners and workers, 2023.

Variables	Categories	Frequency	Percent
Adequate PPEs (*n* = 634)	Yes	27	4.26
	No	607	95.74
Gowns readily available	Yes	55	8.7
	No	579	91.3
Workers wearing PPE during the observation	Yes	25	3.9
	No	609	96.1
Had training on occupational health and safety	Yes	49	7.7
	No	585	92.3
Adequate light	Yes	516	81.4
	No	118	18.6
Adequate space	Yes	490	77.3
	No	144	22.7
Industrial activity supported by a rotatory machine	Yes	442	69.7
	No	192	30.3
Obsolete materials handling	Properly	51	8.0
	improperly	583	92.0
First-aid kit	Not available	627	98.9
	Available	7	1.1

PPE, Personal Protective Equipment; SSI, Small Scale Industries.

### 4.3 Occupational exposure to injury among SSI workers

**Figure 3:**
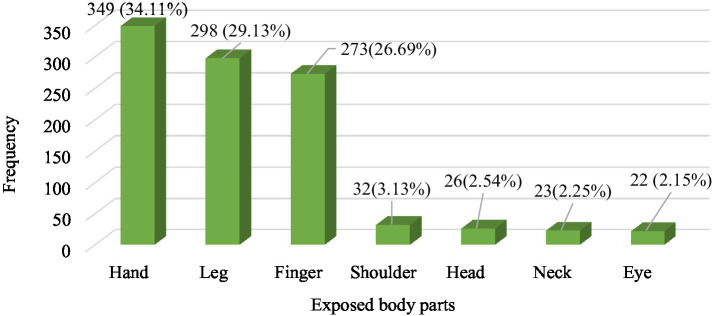
Prevalence (episode) of injuries to exposed body parts, 2023 (*n* = 1,023).

Among the 634 SSI workers, 417 (65.8%) experienced injuries at some point in their careers. Of these, 223 (35.2%) had injuries in the past year, and 182 (28.7%) had injuries in the last month. The most common types of injuries were abrasions (189, 45.3%), cuts (155, 37.2%), burns (56, 13.4%), and fractures (17, 4.1%). Only 58 (13.9%) workers reported their injuries to the relevant authorities; of these, no action was taken in 28 cases (48.3%), 14 (24.1%) received treatment, and 16 (27.6%) were given rest for 2 days. The most frequently injured body parts were hands (349, 34.1%), legs (298, 29.1%), and fingers (273, 26.7%; Figure 3).

**Figure 4:**
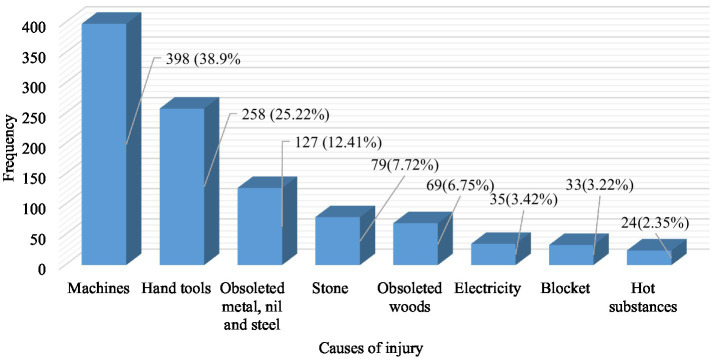
Causes of occupational injury among small-scale industry workers, 2023 (*n* = 1,023).

On the other hand, the current study revealed that the most common causes of injury were machines and hand tools, which accounted for 398 (38.9%) and 258 (25.22%) cases, respectively (Figure 4).

### 4.4 Behavioral-related practice

Among the 634 SSI workers, 363 (57.3%) drank alcohol, 283 of whom drank alcohol 1–3 times a week, whereas 80 (22.04%) drank more than three times. Furthermore, 211 (33.3%) of the SSI workers smoked at least one stick of tobacco cigarettes each day, of which 72 (34.12%), 118 (55.92%) and 9.95% of the workers smoked one to three, 4 to 6 and 7–9 sticks of cigarettes, respectively. On the other hand, 610 (96.2%) SSI workers chewed khat, 598 (97.75%) of whom chewed khat more than twice a week, whereas the remaining 12 (2.25%) chewed khat ≤2 times a week.

### 4.5 Factors associated with occupational related injury among SSI workers

According to the bivariate analysis, SSI workers working in the concrete and metalwork industries were approximately 3.42 times and 1.73 times more likely to be exposed to occupational injury, respectively, than those working in the woodwork industry were. There was a significant association between exposure to injury and the age of the SSI workers, who were in the range of 30–39 years old in comparison to those who were 40–49 years old.

The finding from the multivariate analysis revealed that SSI workers in the concrete industry were approximately 2.41 times more likely (AOR: 2.41, 95% CI: 1.07, 5.46) to be exposed to occupational injuries than those in the woodworking industry were. There was no significant association between occupational injury and age groups; specifically, workers aged 18–29 years (AOR: 1.08, 95% CI: 0.37, 3.18) and 30–39 years (AOR: 1.95, 95% CI: 0.65, 5.82) did not significantly differ from those aged 40–49 years. Workers with no formal education were approximately 2.50 times more likely (AOR: 2.50, 95% CI: 1.01, 2.83) to experience occupational injuries than those with a certificate or higher education. Those who had not received training on occupational health and safety were 1.4 times more likely (AOR: 1.4, 95% CI: 1.35, 3.22) to be injured than those who had received training. Additionally, workers who worked more than 8 h a day were approximately 4.56 times more likely (AOR: 2.88, 95% CI: 2.78, 11.64) to be exposed to occupational injuries than those working 8 h or fewer daily (Table 3).

**Table 3:** Factors associated with occupational exposure to injury among small-scale industry workers working in Harare town, 2023.

Variables (*n* = 634)	Categories	Exposed to injury		COR (95%CI)	AOR (95%CI)	*p* value
		Yes	No			
Types of SSI	Concrete	49	11	3.42(1.67,7.018)*	2.41 (1.07, 5.46)**	0.034
	Construction	138	72	1.47(0.97, 2.22)*	1.13 (0.65, 1.96)	0.662
	Metalwork	131	58	1.73(1.13, 2.67)*	1.59 (0.95, 2.64)	0.076
	Woodwork	99	76	Ref	Ref	
Gender	Female	81	19	2.51 (1.48, 4.27)*	2.01 (1.05, 3.87)**	0.035
	Male	336	198	Ref	Ref	
Age	18–29	234	141	1.227 (0.63, 2.7)	1.08 (0.37, 3.18)	0.887
	30–39	160	59	2.00(1.01, 4.01)*	1.95 (0.65, 5.83)	0.232
	40–49	23	17	Ref	Ref	
Marital status	Single	259	132	2.26 (1.05, 4.95)*	2.04 (0.77, 5.41)	0.151
	Married	145	70	2.39 (1.08, 5.30)*	2.18 (0.80, 5.90)	0.126
	Others	13	15	Ref	Ref	
Educational status	Have no formal education	16	13	2.49 (1.83, 2.75)	2.50(1.01, 2.83)**	0.035
	Elementary school	182	93	1.57 (1.13, 3.17)*	1.57 (0.83, 2.96)	0.162
	Secondary school	136	84	0.53 (0.35, 1.66)	1.89 (1.19, 2.40)**	0.042
	Certificate and above	83	27	Ref	Ref	
Employment condition	Permanent	27	13	Ref		
	Temporally	390	204	1.09 (0.55, 2.15)		
Income in USD	≤36.04	50	23	1.09 (0.48, 2.44)		
	36.5 to 72.1	313	169	0.93 (0.48, 1.81)		
	72.2–108.11	26	11	1.18(0.46, 3.07)		
	>108.11	28	14	Ref		
Service year of SSIs	≤ 3	162	82	0.88 (0.42, 1.8)*	1.62 (0.68, 3.87)	0.650
	4–6	173	110	0.70 (0.34, 1.44)*	1.06(0.455, 2.46)	0.89
	7–9	55	13	1.86 (0.76, 4.67)*	2.66 (0.94, 7.45)	0.064
	≥10	27	12	Ref	Ref	
Service year of employee	≤ 3	314	152	1.17 (0.58, 2.37)		
	4–6	80	52	0.87 (0.41, 1.87)		
	7–9	23	13	Ref.		
PPE	Inadequate	207	400	Ref		
	Adequate	12	15	1.55 (1.51, 3.57)*	2.8 (1.90, 4.26)**	0.002
Had training on OHS?	Yes	14	31	Ref	Ref	
	No	203	386	1.164 (1.6, 2.24)*	1.4 (1.35,3.223)**	0.042
Obsoleted materials	Properly secured	36	15	0.78 (0.42, 1.47)		
	Not properly secured	381	202	Ref		
Chew a khat	Yes	406	204	2.35 (1.03, 5.34)*	1.71 (0.66, 4.43)	0.271
	No	11	13	Ref	Ref	
Smoke cigarette	Yes	141	70	0.93 (0.66, 1.32)		
	No	276	147	Ref		
Drink alcohol	Yes	243	120	0.636 (0.64, 1.23)*	0.85 (0.56, 1.31)	0.471
	No	174	97	Ref	Ref	
Use rotatory machine	Yes	295	147	Ref		
	No	122	70	0.87 (0.61, 1.24)		
Adequate space	Yes	293	197	Ref	Ref	
	No	124	20	4.17 (2.51, 6.91)*	4.6 (2.62, 7.51)**	0.000
Working hour (daily)	≤ 8 h	11	29	Ref	Ref	
	≥ 8 h	406	188	5.69 (2.78, 11.64)*	4.56 (2.88, 9.4)**	0.000
Adequate light	Yes	335	181	Ref	Ref	
	No	82	36	0.81 (0.53, 1.25)*	0.9 (0.65, 1.45)	0.346

OHS, occupational health and safety; PPE, personal protective equipment; SSI, small-scale industry; USD, United States dollar; one USD = 55.5 Ethiopian Birr. *p value of < 0.25.

## 5 Discussion

The current study revealed that 65.8% of small scale industry workers were exposed to occupational injuries, which is higher than the other finding reported 43.2% of workers exposed to occupational injuries (26). This discrepancy may be due to differences in the types of industry included in the study, educational levels, and implementation of health and safety practices.

Among the small scale industry workers exposed to occupational injuries, 35.2% had sustained injuries in the past year. This prevalence is higher than other findings reported in previous studies conducted in Bale, Ethiopia (26), Addis Ababa, Ethiopia (17), and Kampala, Uganda (30), which reported 30, 14.7, and 32.4% prevalence of occupational injuries, respectively. The variation in prevalence of occupational injury may be attributed to differences in occupational health and safety practices, access to or utilization of personal protective equipment, the types of small scale industries, and the characteristics of the study populations.

Furthermore, current study revealed that the most common body parts exposed to occupational injury were the hands, legs, and fingers, which accounted for 349 (34.11%), 298 (29.13%), and 273 (26.69%) exposure episodes, respectively. However, another study conducted in Addis Ababa, Ethiopia, reported that the prevalence of leg and finger/hand injuries among construction workers was 46.6 and 43.5%, respectively (31). The variation in the prevalence of occupational injuries among different body parts may be attributed to the workload, behavior-related factors, supportive supervision, or types of material or devices employed to implement the activities.

Furthermore, the current study revealed that 601 (94.8%) SSI workers did not use personal protective equipment, which was higher than the findings reported by a study conducted in Hawassa town, Ethiopia, which reported 57.4% of the workers did not access or lack PPE during their work (14). The difference in the utilization of personal protective equipment may be due to the variation in working environment-related factors, inadequate awareness, and lack of supervision on occupational health and safety The current study revealed that 594 (93.7%) small scale industry workers worked more than 8 h daily, which was significantly greater than the findings of another study conducted in Addis Ababa, Ethiopia, which reported that 8.1% of the employees had working less than 8 h per day (31). Furthermore, the current study revealed that only 49 (7.7%) SSI workers had training on occupational health and safety, which was lower than the findings reported from Addis Ababa City in 2015, which report 16.0% of employees had received workplace safety training (31). These differences may be due to variations in the implementation of occupational health and safety (OHS) practices and the availability of supportive supervision from both governmental and nongovernmental organizations.

The findings from multivariate analysis revealed that small scale industry workers working in the concrete industry were approximately 2.41 times (AOR: 2.41, 95% CI: 1.07, 5.46) more likely to be exposed to occupational injury, whereas those working in the metal industry were 1.59 times (AOR: 1.59, 95% CI: 1.02, 2.64) more likely to be exposed to injury than woodwork industry workers. The findings of the present study were in line with those of another study conducted in Mekele City, Ethiopia, in 2013, which reported that being a metal worker [AOR: 3.17, 95% CI: 2.07–4.85] or a woodworker [AOR: 2.34, 95% CI: 1.39–3.92] was associated with high prevalence of occupational injury. Furthermore, the findings of the current study are supported by another study conducted in Addis Ababa, Ethiopia, that reported an association between occupational injury and job categories (AOR: 3.52, 95% CI: 1.08, 11.41) (17). In general these findings highlight that the concrete and metal industries have greater risks of occupational injuries than the other industries. The increased risk may be due to different factors such as the nature of the work, implementation of occupational health and safety measures, and the overall working environment.

In addition, this study revealed that small scale industry workers working in industries with inadequate space are approximately 4.6 times (AOR: 4.6, 95% CI: 2.62, 7.51) more likely to be exposed to occupational injury than those working in industries with adequate space. This finding was in line with the finding from another study conducted in Addis Ababa, Ethiopia, which reported a significant association (AOR: 3.85; 1.14, 13.04) between inadequate work space and occupational injury among SSI workers (17).

According to the current study, small scale industry workers who had training or orientation toward occupational health and safety were 1.4 (AOR: 1.4, 95% CI: 1.35, 3.22) times more likely to be exposed to injury than those who had no training. This finding was supported by the findings reported by another study conducted in Ambo town, Ethiopia, which reported a significant association between occupational injury and training occupational health and safety (AOR: 2.05, 95% CI: 1.26–4.37) (27).

Small scale industry workers working more than 8 h daily are 4.56 times (AOR: 4.56, 95% CI: 2.88, 9.4) more likely to be exposed to occupational injury than those working less than 8 h daily. The finding of this study is in line with another finding reported by a study conducted in Mekele City, Ethiopia, which reported an association between occupational injury among small scale industry workers and workload [AOR = 2.73, 95% CI: 1.92–3.87].

In general, this study revealed that a significant proportion of small scale industry workers in Harare town experienced occupational injuries, with a notably high prevalence of injury. The findings highlight critical gaps in occupational health and safety practices, including the need for improved PPE utilization, enhanced safety training, and better workspace conditions. Addressing these issues could significantly reduce the risk of occupational injuries among SSI workers.

### 5.1 Limitation of the study

The current study mainly focused on the survey results aimed at determining the prevalence of occupational injuries among small-scale industry workers, and not addressed laboratory analysis. Therefore, the authors of this work recommend further studies that incorporate the laboratory analysis or experimental studies, particularly to determine the level of exposure for different hazards and assess the health risk. The future researcher should also consider how the cognitive challenges, emotional states, and work stress influence occupational health and safety practice as well as occupational injuries.

## 6 Conclusion

The current study revealed that more than 6 out of 10 small-scale industry workers were exposed to injuries during their career time, of which more than one-third of small-scale industry workers were exposed to occupational injuries in the last year. Furthermore, this study found a statistically significant association between occupational injuries and lower educational status, having no training, poor working conditions, and higher workloads. Therefore, the study highlights the need to implement occupational health and safety practices, including targeted training for small scale industry workers and supportive supervision, to reduce the risk of occupational injuries.

## Data Availability

The original contributions presented in the study are included in the article/supplementary material, further inquiries can be directed to the corresponding author.
